# Waning immunity six months after BioNTech/Pfizer COVID-19 vaccination among nursing home residents in Zagreb, Croatia

**DOI:** 10.3325/cmj.2021.62.630

**Published:** 2021-12

**Authors:** Branko Kolarić, Andreja Ambriović-Ristov, Irena Tabain, Tatjana Vilibić-Čavlek

**Affiliations:** 1 Andrija Stampar Teaching Institute of Public Health, Zagreb, Croatia; 2 Faculty of Medicine, University of Rijeka, Rijeka, Croatia; 3 Ruđer Bošković Institute, Zagreb, Croatia; 4 Croatian Institute of Public Health, Zagreb, Croatia; 5 University of Zagreb School of Medicine, Zagreb, Croatia

## Abstract

**Aim:**

To assess the humoral immunity to COVID-19 in nursing home residents six months after vaccination.

**Methods:**

This seroepidemiological research enrolled 118 residents of one nursing home in Zagreb. All participants received two doses of BioNTech/Pfizer COVID-19 and had no previously detected SARS-CoV-2 infection. The samples were tested for the presence of neutralizing antibodies using a virus neutralization test. A SARS-CoV-2 strain isolated in Vero E6 cells from a Croatian COVID-19 patient was used as a stock virus. Neutralizing antibody titer was defined as the reciprocal of the highest serum dilution that showed at least 50% neutralization. Neutralizing antibody titer ≥8 was considered positive.

**Results:**

Sixty-four (54%) participants had a positive neutralizing antibody titer, 27 (23%) had a low positive titer (titer 8), and 27 (23%) had a negative titer. Women had a significantly higher median titer than men (16 [interquartile range, IQR 24] vs 8 [IQR 12], Mann-Whitney U = 1033, *P* = 0.003). Age was negatively but not significantly correlated with neutralizing antibody titer (Spearman’s rho -0.132, *P* = 0.155).

**Conclusion:**

Almost half of the participants (46%) had a negative or low positive titer six months after having been fully vaccinated. This study suggests that humoral immunity among nursing home residents considerably wanes six months after BioNTech/Pfizer COVID-19 vaccination. Our results could contribute to the discussion about the need for a booster dose.

By October 2021, more than 238 million coronavirus disease (COVID-19) cases were confirmed and around 4.9 million deaths recorded across more than 200 countries (1). COVID-19, caused by severe acute respiratory syndrome coronavirus 2 (SARS-CoV-2), has posed a serious threat to public health systems. In many European countries, COVID-19-related deaths of nursing home residents contribute to more than one third of all COVID-19-related deaths (2). The high morbidity and mortality observed among residents in long-term care facilities are a major challenge for disease prevention and control in such settings (3).

The most effective intervention for preventing the spread of infectious diseases is vaccination. Various SARS-CoV-2 vaccine types have been developed, including mRNA vaccines, adenovirus-based vector vaccines, DNA vaccines, inactivated vaccines, and recombinant subunits vaccines. All vaccines so far approved in the European Union are either mRNA vaccines using lipid nanoparticles as vectors for mRNA delivery or adenovirus-based vector vaccines. All these vaccines target the spike protein, which is the main antigen component of SARS-CoV-2 structural proteins (4,5).

Humoral immunity acts as an important part of immunity against viral infection, mainly through the production of neutralizing antibodies against viruses. Neutralizing antibodies play a critical role in controlling SARS-CoV-2 infection (6). In addition, the presence of each SARS-CoV-2-specific CD4+ and CD8+ T cells was associated with a milder disease (7). However, there has been much controversy over the role of humoral immune response in COVID-19, including the dynamics of antibody response, correlation with disease severity, and duration of neutralizing antibodies and memory B-cell response (8).

Recent studies have shown that the neutralizing antibody level highly predicts immune protection. Croatia started mass vaccination against SARS-CoV-2 on December 27, 2020. Nursing home residents have been prioritized due to a high case fatality. After vaccination, restrictive counter-epidemic measures introduced in nursing homes were eased. However, the number of infected nursing homes residents has recently increased, prompting a discussion about the need for a booster dose. This study aimed to assess the extent of waning immunity in this population by measuring neutralizing antibody titers in one nursing home to assess the need for a third vaccine dose.

## Patients and methods

This seroepidemiological research enrolled 120 residents of one nursing home in Zagreb. Of 607 residents, 417 were women. The inclusion criteria were age over 64 and full vaccination (two doses) against COVID-19. The only exclusion criterion was previously diagnosed COVID-19. All participants were vaccinated at the same time. They received the first dose of BioNTech/Pfizer COVID-19 in December 2020 and the second dose in January 2021. The samples were collected in the last week of July 2021, six months after the second vaccination. The participants gave oral informed consent; data and samples were anonymized to protect the participants' identities. The study was approved by the Ethics Committee of Andrija Stampar Teaching Institute of Public Health.

The initial serological screening for SARS-CoV-2 binding IgG antibodies was performed using a commercial enzyme-linked immunosorbent assay based on recombinant S and nucleocapsid protein antigens of SARS-CoV-2 (Vircell Microbiologists, Granada, Spain). All samples were further tested for the presence of neutralizing antibodies using a virus neutralization test (VNT). A SARS-CoV-2 strain isolated in Vero E6 cells from a Croatian COVID-19 patient was used as a stock virus. Virus titer (TCID_50_) was calculated using the Reed and Muench formula. Neutralizing antibody titer was defined as the reciprocal of the highest serum dilution that showed at least 50% neutralization. Neutralizing antibody titer <8 was considered negative and that of ≥8 was considered positive (9). Only VNT results are presented.

### Statistical analysis

Data are presented as counts with percentages or medians with interquartal ranges. Differences between the sexes were assessed with the Mann-Whitney test, and the correlation between age and neutralizing titer with the Spearman’s rho test. Logistic regression was used to assess the strength of association between VNT seropositivity and sex, while controlling for age as a biological confounder. Statistical analysis was performed with STATA/MP 17 (StatCorp LLC, College Station, TX, USA).

## Results

Two participants were excluded due to a previous COVID-19 infection, which left 118 samples in the final VNT analysis. The tested group consisted of 79 (67%) women. The median age was 82.5 years (interquartile range [IQR] 10.25).

**Figure 1:**
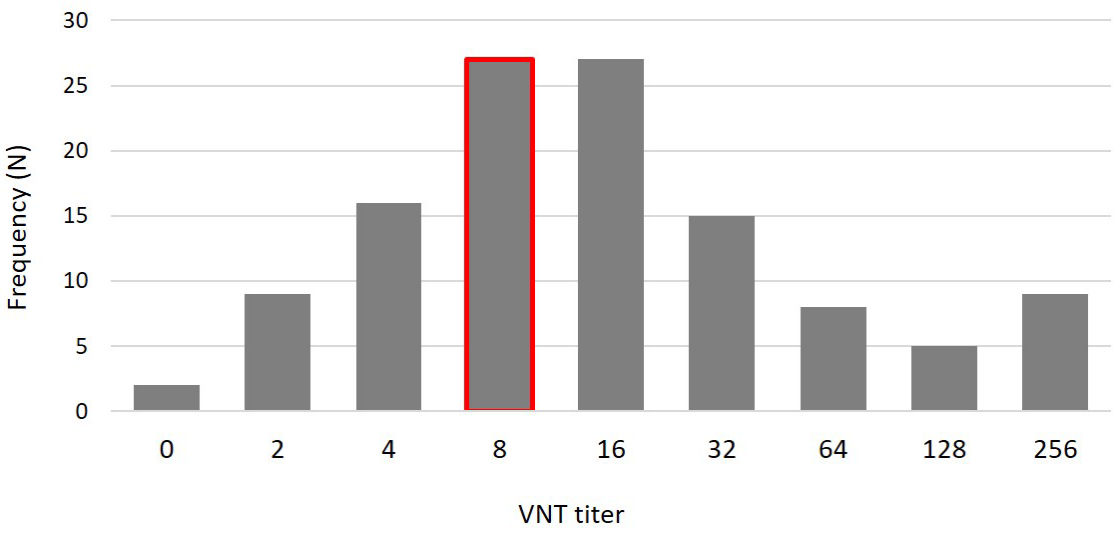
Distribution of participants according to virus neutralization test titer (the cut-off titer is marked red).

**Figure 2:**
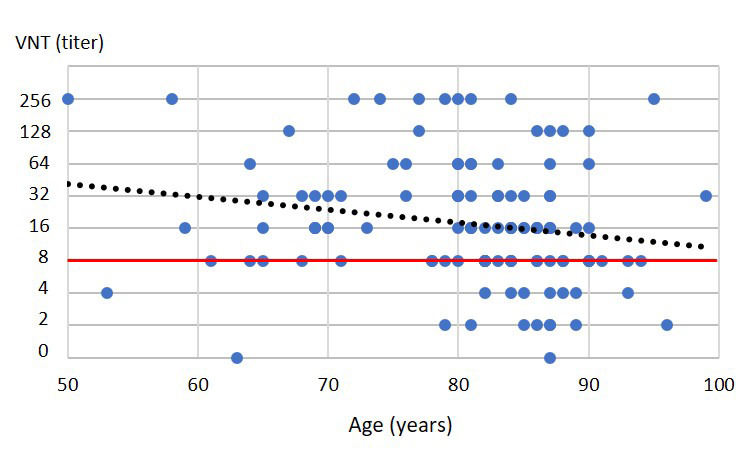
The correlation of age and neutralizing antibody titer (the cut-off titer is marked red).

Neutralizing antibody titers are presented in Figure 1. Sixty-four (54%) participants had a positive neutralizing titer, 27 (23%) had a low positive neutralizing titer (titer of 8), and 27 (23%) had a negative neutralizing titer. The median neutralizing antibody titer was 16 (IQR 24) for women and 8 (IQR 12) for men, with the difference being significant (Mann-Whitney U 1033, *P* = 0.003). After age adjustment, women still had significantly higher odds for having a positive neutralizing antibody titer (odds ratio 5.04, 95% confidence interval 1.95-13.01). Age negatively, but not significantly, correlated with neutralizing antibody titer (Spearman’s rho -0.132, *P* = 0.155) (Figure 2).

## Discussion

In the present study, almost half of the participants (46%) had a negative or low positive neutralizing antibody titer. A trend of declining humoral response after vaccination, more pronounced in the age group >65 years, has been shown by several authors (10-13). In addition to immune senescence, aging may be associated with a reduced functional antibody activity, even in the presence of normal antibody concentrations (11).

An interesting finding in this study is a higher neutralizing antibody titer in women compared with men. Among Italian health care workers, women also had a significantly higher serological response after vaccination (14). A study from the United Kingdom identified four vaccine responder subgroups, including a “low responder” group, consisting mostly of people aged >75 years, men, and individuals with long-term health conditions (15).

Although some studies found a strong negative correlation between older age and antibody concentration after vaccination (11,16), this study found a weaker and non-significant correlation. Several limitations of the study could affect our conclusions. Although only residents who reported no history of COVID-19 were enrolled, some of the participants might have had asymptomatic or mild infection and had not been tested with reverse transcriptase polymerase chain reaction test. In addition, as serological testing was not performed a few weeks after vaccination, a certain number of residents might have been non-respondents.

Despite these limitations, our study suggests a considerable waning of humoral immunity among nursing home residents six months after BioNTech/Pfizer COVID-19 vaccination. Decision makers should consider introducing a booster dose to decrease the risk of infection in the most vulnerable groups. However, further studies on a large sample are needed to confirm our findings.
